# Custom-designed orthopedic implants evaluated using finite element analysis of patient-specific computed tomography data: femoral-component case study

**DOI:** 10.1186/1471-2474-8-91

**Published:** 2007-09-13

**Authors:** Ola LA Harrysson, Yasser A Hosni, Jamal F Nayfeh

**Affiliations:** 1 Department of Industrial and Systems Engineering, North Carolina State University, Campus Box 7906, Raleigh, USA; 2 Department of Industrial Engineering and Management Systems, University of Central Florida, Orlando, USA; 3 Department of Mechanical, Materials and Aerospace Engineering, University of Central Florida, Orlando, USA

## Abstract

**Background:**

Conventional knee and hip implant systems have been in use for many years with good success. However, the custom design of implant components based on patient-specific anatomy has been attempted to overcome existing shortcomings of current designs. The longevity of cementless implant components is highly dependent on the initial fit between the bone surface and the implant. The bone-implant interface design has historically been limited by the surgical tools and cutting guides available; and the cost of fabricating custom-designed implant components has been prohibitive.

**Methods:**

This paper describes an approach where the custom design is based on a Computed Tomography scan of the patient's joint. The proposed design will customize both the articulating surface and the bone-implant interface to address the most common problems found with conventional knee-implant components. Finite Element Analysis is used to evaluate and compare the proposed design of a custom femoral component with a conventional design.

**Results:**

The proposed design shows a more even stress distribution on the bone-implant interface surface, which will reduce the uneven bone remodeling that can lead to premature loosening.

**Conclusion:**

The proposed custom femoral component design has the following advantages compared with a conventional femoral component. (i) Since the articulating surface closely mimics the shape of the distal femur, there is no need for resurfacing of the patella or gait change. (ii) Owing to the resulting stress distribution, bone remodeling is even and the risk of premature loosening might be reduced. (iii) Because the bone-implant interface can accommodate anatomical abnormalities at the distal femur, the need for surgical interventions and fitting of filler components is reduced. (iv) Given that the bone-implant interface is customized, about 40% less bone must be removed. The primary disadvantages are the time and cost required for the design and the possible need for a surgical robot to perform the bone resection. Some of these disadvantages may be eliminated by the use of rapid prototyping technologies, especially the use of Electron Beam Melting technology for quick and economical fabrication of custom implant components.

## Background

Conventional knee and hip implants have been successfully used for over 30 years and are the two most common orthopedic implant surgeries performed around the world. According to public information, between 250000 and 300000 total knee arthroplasties (TKAs) are performed each year in the United States alone; and the number is rapidly increasing. In many cases, conventional knee implants provide a satisfactory result that brings the patient back to a near-normal and active lifestyle. However, in some cases, standard implant components are not sufficient because of abnormal joint anatomy or postoperative complications [1,2].

In such cases a custom-designed implant system is necessary and is most often based on a patient-specific Computed Tomography (CT) data set. Conventional knee implant components are used as the base, and the bone-implant interface is customized to fit the specific patient.

In a recent study, it was concluded that younger patients have a lower success rate than older patients when standard implant components are used [3]. According to the study, aseptic loosening is the most common cause for premature failure in younger patients, which is in agreement with previous studies [4]. It has been suggested that a more active lifestyle in younger patients is the major cause for premature failure. For this reason, many countries try to delay the surgery until the patient has reached the age of 65 [5].

Loosening of the components is usually caused by micromotions that prevent appropriate bone ingrowth or bone remodeling due to uneven stress distribution on the bone surface [6]. The uneven stress distribution is caused by a bone-implant interface design that has been restricted by the surgical techniques currently available. The bones are reshaped to fit the implant components by planar cuts using an oscillating saw and cutting guides. The resultant bone shape is squared-off, rather than rounded as is its original shape. Thus, the forces generated due to the patient's weight and activities are distributed in such a way that the newly created "corners" of the distal femur take a disproportionate amount of stress, rather than the forces being evenly distributed over the rounded ends of a natural femur. This can lead to bone remodeling and loosening of the prosthetic joint.

The proximal tibia is reshaped using a single planar cut, and the tibial tray is normally secured using a stem configuration. While loading the tibial tray, high stress concentrations are caused by the stem; the cancellous bone can collapse, leaving a void around the stem. If the tibial component is not properly sized and if the tibial component does not have enough cortical bone supporting it, then the component can protrude into the cancellous bone and create an implant failure [7].

The Ultra High Molecular Weight Polyethylene (UHMWPE)-bearing component can also cause failures leading to revision surgery and replacement of all components. In many cases, the bearing surface is completely worn through; and metal-on-metal contact between the femoral component and the tibial tray causes discomfort and loss of motion. In other cases the wear particles cause osteolysis; and a revision surgery is required to address the pain, discomfort, and lack of mobility. Further, it has been found that micromotion between the tibial tray and the UHMWPE-bearing component increases the wear rate and significantly reduces the component's longevity [8].

Many other factors (such as loosening of femoral and tibial components, sacrificed or torn ligaments, and mobile bearing components) can increase the wear rate of the bearing surface, which will decrease the component's longevity and increase the risk for osteolysis [9-12]. Conventional knee implant components are based on a generic joint anatomy, while all patients are unique. In many cases, the gait of the patient changes after a TKA; and proper walking and ambulation has to be relearned. Owing to the generic shape of the femoral component and the patellar groove, it is common to resurface the patella in order to prevent dislocation, even though the patella is not affected by osteoarthritis. Studies have shown that resurfacing of a healthy patella can cause unnecessary postoperative anterior pain for the patients [13]. According to the same study, a correctly designed femoral component with a sufficient patellar grove can avoid the resurfacing and reduce the risk for postoperative pain. Today, many implant companies offer implant components that are customized according to size and shape.

This paper will provide a new proposed customized knee implant system that could provide a better result for younger patients and patients with an abnormal joint anatomy. The proposed custom design process can be used for a wide variety of implants and is not restricted to knee-implant components. The rest of this paper is organized as follows. In the second section we describe the design of the femoral component, including the required Finite Element Analysis. In the third section we summarize our results, showing the stress distributions under different load conditions. In the fourth section we summarize the main conclusions of this work.

## Methods

Currently, most custom knee implants are designed using a generic shape of the articulating surfaces; and the bone-implant interface is created using planar cuts. The customization is normally limited to better sizing and fit, since this is very important for the longevity of the implant. In some cases the bone-implant interface is customized to accommodate joint deformities. In recent years, robotic systems have been developed for use in orthopedic surgery [14,15]. The robot uses an end mill to machine the bone to fit the implant, and the tool path is generated based on a CT-derived Computer Aided Design (CAD) model of the joint and a CAD model of the implant. It has been proven that a robot can achieve a much more precise cutting operation than the average orthopedic surgeon can achieve using hand tools and cutting guides. According to a study by Toksvig-Larsen et al. [16], the average contact surface between the bone and the implant is only about 50% when using conventional methods, which is not sufficient for a cementless implant to ensure prompt and secure fixation. The robots, on the other hand, can achieve an average bone-implant contact surface of 95%, which reduces the fixation time and improves the initial stability of the implant. A robot is capable of performing cutting operations of complex freeform surfaces and is not limited to planar cuts, as is the case when using conventional cutting methods. To take full advantage of the robotic capabilities and to attempt to solve some of the problems with the current knee implant designs, a new custom knee implant design is proposed.

### Custom design

The custom design phase was initiated by the acquisition of a Computed Tomography (CT) scan of the patient's knee joint. The image data was imported into Mimics version 8.1.1 (Materialise, Leuven, Belgium) for editing and three-dimensional (3D) reconstruction. The resolution of the CT images and the slice distance will affect the accuracy of the model. For this project, a CT scan with an XY-resolution of 512 × 512 pixels was used, with a resulting pixel size of 0.391 mm and the helical scan was retroreconstructed into 1 mm slices. The total number of slices in the scan was 217, and the scan was performed using 0° gantry tilt.

**Figure 1:**
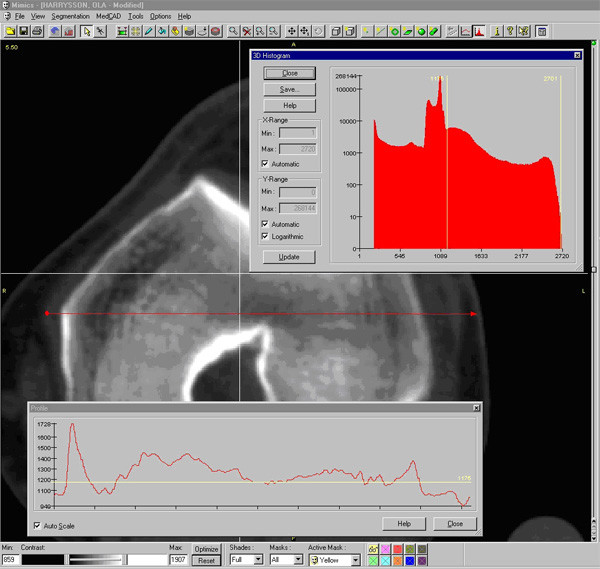
Profile line is used to determine the appropriate threshold for the 3D reconstruction.

Once the CT images were imported into Mimics, the first step was to adjust the contrast for easy viewing, followed by selection of an appropriate threshold value for the region growing function. To select an appropriate threshold, the profile line function was used, which measures the density of the tissue along a user-defined line based on the grayscale values (Figure 1). The selection of the threshold value is crucial for the accuracy of the resulting model. If the value is selected too low, then the resulting model will be smaller than the actual knee joint, and vice versa. The threshold function will separate the soft tissue from the hard tissue isolating the bone structure only.

To complete the isolation of the hard tissue, the region growing function was used. This function connects all volumetric pixels (voxels) within the threshold that are physically connected to the initially selected voxel. Because the distal femur, the proximal tibia, and the patella are not connected, multiple region growings were applied using different masks and colors. Each mask was converted into a 3D model using the "*calculate 3D*" function. Because of the thresholding function, some of the cancellous bone was not included; and this created unwanted internal voids in the model. A complete solid model was desired for the custom design phase, and editing of the masks was necessary. Filling of the voids was accomplished by using several editing techniques like cavity fill, draw, and local thresholding.

**Figure 2:**
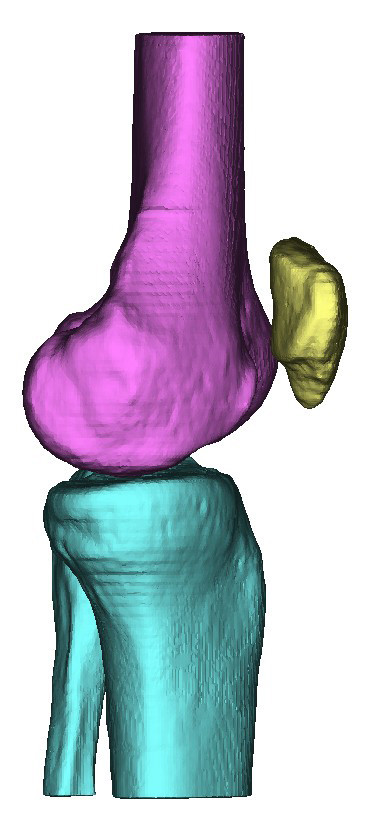
Initial 3D computer model of the knee joint produced using Mimics by Materialise, Belgium.

Mimics uses a smoothing algorithm during the 3D reconstruction phase to create a more realistic model. However, the resulting 3D models showed ridges that coincided with the image slices where the cross-sectional area changed significantly from one image to the next (Figure 2). Further, the very distal surface of the femoral condyles and the very proximal surface of the tibia were flat because of missing information from one slice to the next. These model defects could be corrected using a different software package. Unfortunately Mimics does not currently have the ability to export the 3D-model into a CAD format that can be manipulated by standard CAD packages.

The most efficient method for the required data manipulation was to convert the 3D-model into a stl-file format (i.e., a format designed for stereolithography) that could be converted into a 3D CAD format by another software package. The stl-file format is a triangular surface mesh used by the rapid prototyping industry as a standard file format. An stl-file generated by Mimics based on the mask information contains a large number of triangles with various sizes and shapes. For the finite element analysis, the mesh will be based on the stl-file's triangles, which need to be of equal size and shape. The conversion of the stl-file into a CAD-file format can become a cumbersome task because of the uneven triangular mesh. To enhance the stl-file prior to further conversions, the remesh module in Mimics was used. The remesh module is based on a set of algorithms with user-specified parameters that will reshape and resize the triangles through a user-defined number of iterations. After the remesh was completed, the number of triangles had been significantly reduced and the triangular mesh was even in size and shape.

Geomagic Studio V7.0 (Raindrop Geomagic, Triangle Park, NC) was used to convert the stl-file into a NURBS (Non Uniform Rational B-Spline) format. Geomagic Studio uses the triangular mesh to create NURBS surfaces that can be exported as a solid CAD model using a STEP-file format. The basic idea is to use the CAD model of the femur and the tibia as a base for the custom-designed components.

### Design of femoral component

The articulating surface of a conventional knee implant is of generic shape and causes the problems mentioned earlier. Most patients' gaits are altered due to the change in distal femur geometry. The ease of adapting to the new gait can vary widely but does present a problem for many patients. Further, most patients need to have their patella resurfaced because of the change in the patella grove geometry. If the articulating cartilage on the patella is affected by arthritis, then the resurfacing is necessary to control the pain. If the cartilage is not affected by arthritis, then the resurfacing is solely done to prevent patella dislocation because of the mismatch in geometry. The patella resurfacing is an additional surgical intervention that requires an implant component as well, but a more important consideration is that many patients suffer from postoperative pain due to the procedure [13]. Emoto et al. [13] suggest that the patellar resurfacing could be eliminated in most cases for patients without an arthritic patella if the grove on the femoral component was more closely customized for the patient's patella.

**Figure 3:**
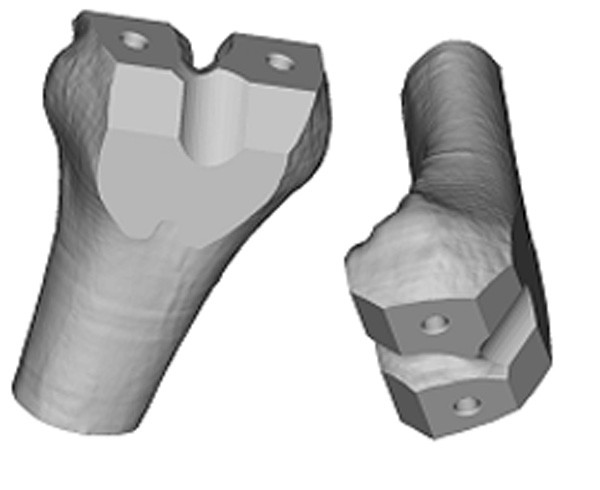
A 3D computer model of a distal femur cut to fit a conventional femoral component.

As mentioned in the introduction, the conventional femoral components have a very simple bone-implant interface of five flat surfaces. The sharp edges on the bone create stress concentrations under load, which will lead to bone remodeling and an increase in bone density. The flat areas between the sharp edges are stress shielded, which will lead to bone loss and loosening of component (Figure 3).

The proposed custom femoral component design presented in this article has addressed all the above problems. The CAD model of the distal femur derived from the CT images was used as the base for the implant design. To address the problems with gait change and patellar resurfacing, the original shape of the articulating surface can be preserved. If the arthritis has progressed beyond the cartilage, damaging the bone as well, then this can be repaired in the software prior to converting the model into a CAD format. If bone deformities are present causing abnormal joint anatomy, then this can also be compensated for using the editing tools in the software. To take full advantage of the new robotic surgical tools, we propose optimizing the bone-implant interface to minimize the uneven stress distribution that can result in bone remodeling and implant loosening. Surgical robots are currently used to perform the standard cutting procedures, but much more complex surfaces can be achieved. The idea is to design an implant that minimizes the amount of bone to be removed and provides an even stress distribution on the bone-implant interface. To even out the stress distribution, the interface surface should have a geometry similar to that of the distal femur, but without any undercuts to enable easy implantation.

**Figure 4:**
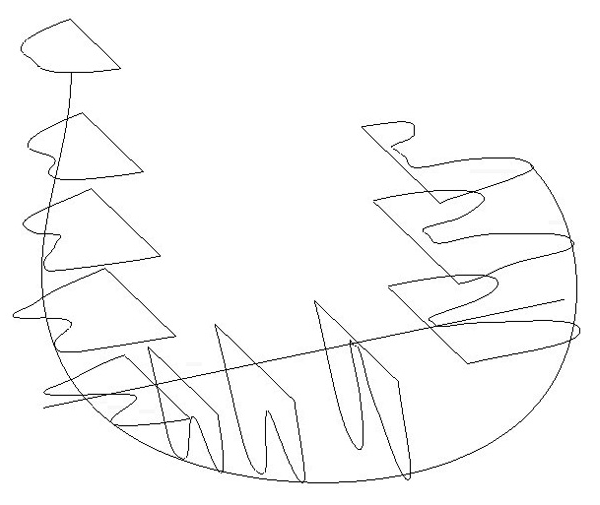
Spline curves used to create the bone-implant interface surface.

The proposed custom implant design was done using Pro/ENGINEER (PTC, Needham, MA). A CAD model of the patient's distal femur was imported and used as the base for the design. The articulating surface was kept intact to avoid altering the gait and to preserve the correct patellar grove so as to prevent resurfacing and patellar dislocation. The bone-implant interface surface was designed to mimic the articulating surface but without undercuts. A set of spline curves were created along the interface surface in a radial pattern (Figure 4), and a single spline was created to connect all curves in a central plane between the condyles.

**Figure 5:**
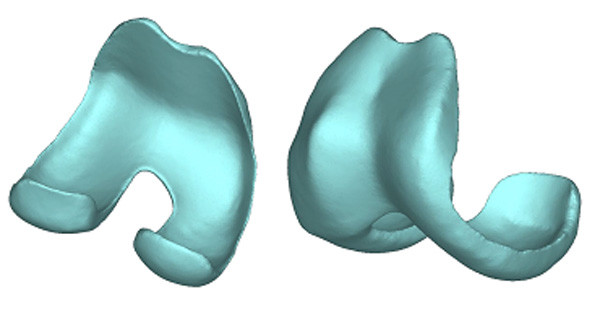
3D computer model of a custom designed femoral component.

A swept-blend command was used to create the smooth articulating surface. The bone-implant interface was created using a new set of curves in the same planes as the original curves. The inner curves were offset from the original curves and then manually edited to avoid any undercuts. The offset distance was kept uniform to create an implant with constant wall thickness. A new center plane curve was created as well to prevent the need for undercuts. A swept-blend-cut command was used to create the bone-implant interface. Additional cuts and fillets were added to provide an implant with smooth surfaces and edges (Figure 5).

The result was a femoral component with a natural articulating surface and a bone-implant interface that more evenly distributes the load on the bone surfaces. The thickness of the implant could be adjusted based on material choice (titanium or cobalt-chromium) as well as patient weight and activity level.

### Finite element analysis (FEA)

To test the hypothesis that the proposed bone-implant interface surface will provide more even stress distribution, finite element analyses were performed.

To reduce the computational effort, two implants were designed with the same simple articulating surface. One bone-implant interface surface was designed with the conventional five flat surfaces; and the other bone-implant interface was designed with the proposed custom smooth surface. Distal femur models for the FEA were created by aligning the implants to a distal femur model and by using a Boolean operation (distal femur minus femoral component) to simulate the bone-cutting procedure. ABAQUS 6.4-3 CEA (ABAQUS, Inc. Providence, RI) was used to perform the FEA, and the tetrahedral mesh was based on the triangular surface mesh generated by Mimics.

**Figure 6:**
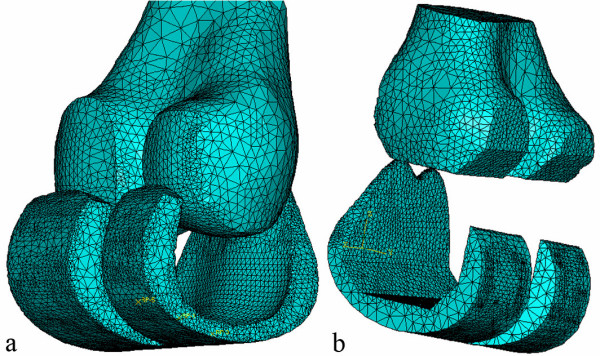
a) 3D computer model of a custom femoral implant component and a cut distal femur b) 3D computer model of a standard femoral implant component and a cut distal femur.

To enhance the original triangular surface mesh, the remesh module in Mimics was used. By using several remeshing algorithms and user-specified parameters, the number of triangular elements can be reduced and reshaped to provide an even surface mesh. If too many triangles are used, then the computation becomes very time consuming and computer intensive. The remeshing of the distal femur and the implants must be performed prior to using the Boolean operation. Table 1 summarizes the number of tetrahedral elements that were used for each model. Figure 6A shows the finite element mesh of the custom implant and distal femur. Figure 6B shows the finite element mesh for the standard implant and distal femur.

**Table 1:** Number of tetrahedral elements used for each model

**FEA model**	**Number of tetrahedral elements**
Custom femur	25521
Custom implant	61858
Standard femur	19657
Standard implant	49724

**Figure 7:**
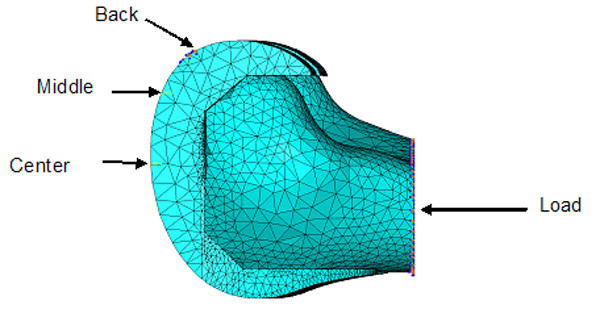
Load and constraints used for all finite element analysis models.

The femoral models where divided into two sections for material assignment. An outer shell of cortical bone was defined, and the inner remaining material was specified as cancellous bone. The majority of the bone-implant interface is cancellous bone with a thin rim of cortical bone around the edges. The material properties used for the analysis are specified in Table 2. Three positions out of a gait cycle were used for the analyses (Figure 7). The center position for the reaction force represents a standing position or starting position of a gait cycle. The middle position represents the end of a normal walking gait cycle, and the back position represents an extreme position for climbing stairs. The angle of the load was adjusted for the middle and back position to better simulate the correct conditions. Load levels for the center, middle, and back positions were 2200 N, 3200 N, and 2800 N, respectively [17]. The boundary conditions were defined as narrow surfaces going across the implant of an approximately width of 2–3 mm. This represents the limited surface contact between the femoral component and the tibial tray.

**Table 2:** Material properties used for analysis [18]

**Material**	**Modulus of elasticity**	**Poisson ratio**
Cancellous bone	0.4 Gpa	0.3
Cortical bone	15 Gpa	0.46
CoCr implant	220 GPa	0.3

The interaction between the distal femurs and the implants was defined as standard augmented Lagrange contact without separation after contact. Default stiffness was used for the contact calculation. Finite sliding was allowed and only overclosed nodes were adjusted. The femurs were fixed in the contact step and deactivated for loading. The finite element analyses were solved using a blade server in the High Performance Computing Center at North Carolina State University.

## Results

**Figure 8:**
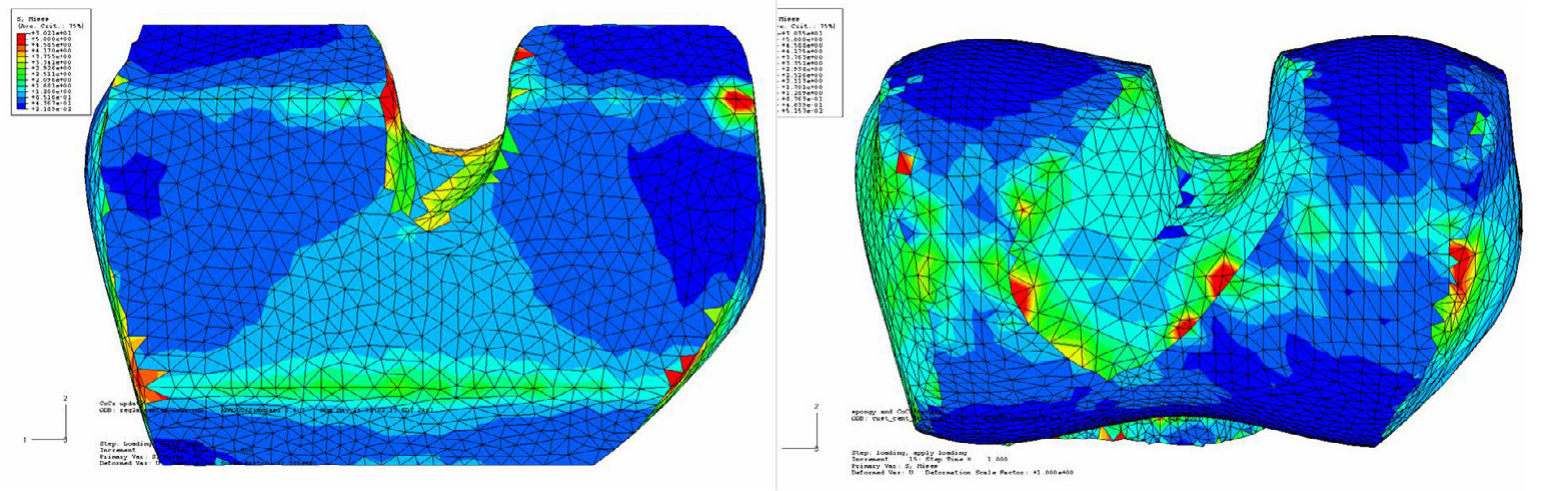
**Comparison of stress distribution on bone surface for conventional and custom implant with loading and reaction force in center location**. Maximum stresses are shown in red color at a level above 5 MPa. Green contour stress levels are 2.5 MPa.

All FEA plots were done at the same stress scale level. All stresses plotted were Von Mises. Figure 8 shows the result of the first load case for both the conventional and custom implant design (center position). Both bones were loaded identically according to the specification in the subsection describing the finite element analysis. The conventional bone interface showed stress concentrations along the sharp edges, while the custom implant showed a more uniform stress distribution. The level of the contact surface stresses and the stress distributions for the conventional bone interface were in the same range as previous studies [17,18]. For the conventional implant, the center position stresses were on average 2.17 MPa at the sharp edges; and for the custom implant, the center position stresses were on average 2.53 MPa in the contact regions. The average stresses at the contact edges for the conventional implant were 1.59 MPa and 1.3 MPa for the back and middle positions, respectively.

**Figure 9:**
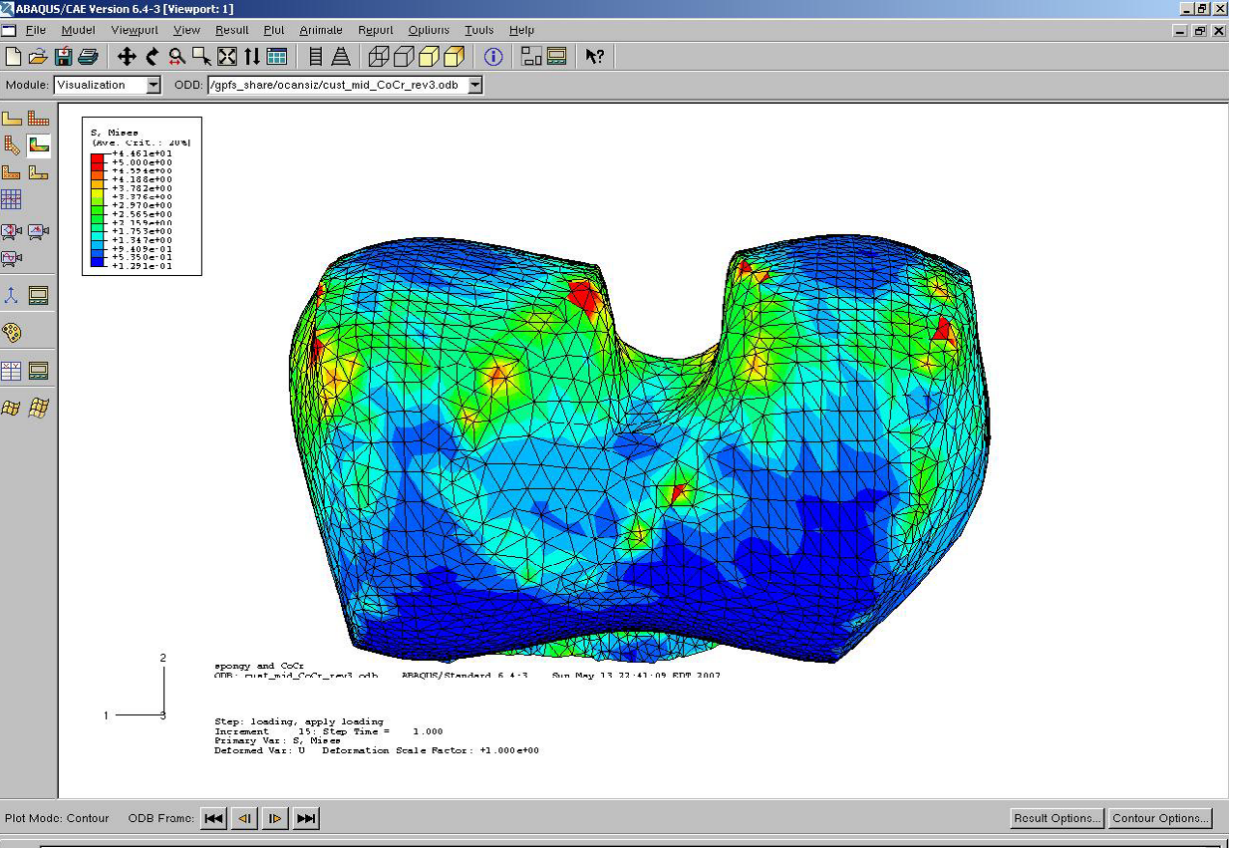
**Bone interface of the custom implant under second condition showing even stress distribution across both condyles**. Stresses are between 0.9 MPa to 3 MPa on the contact surface.

**Figure 10:**
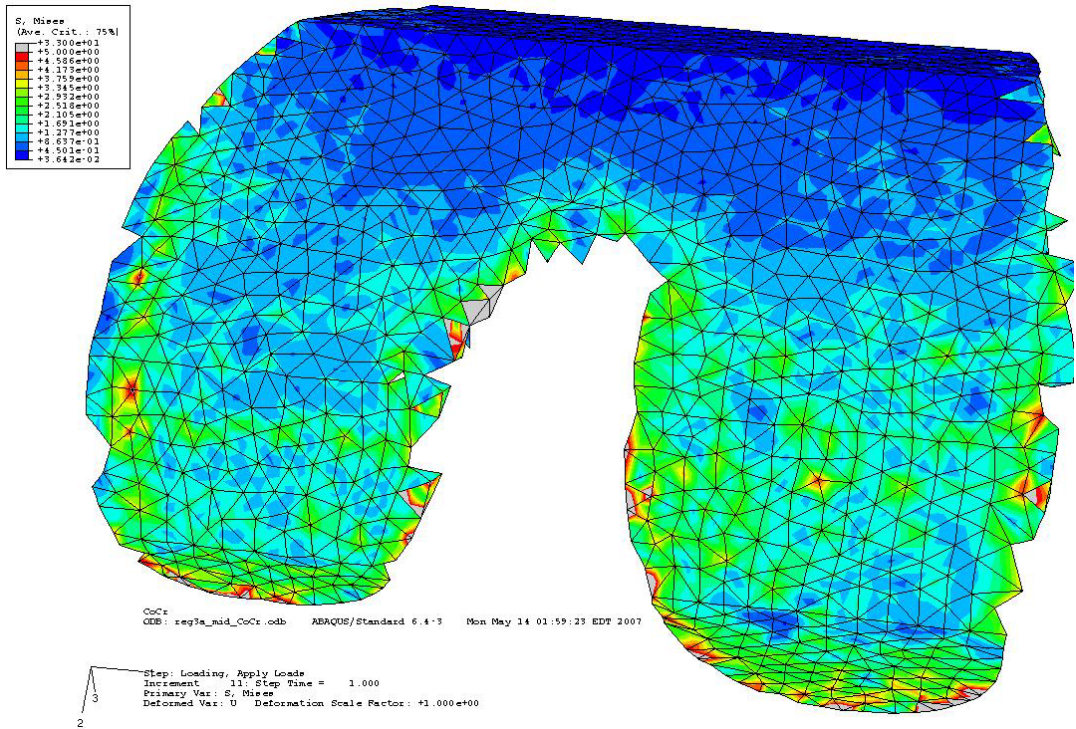
**Bone interface of the conventional implant under third load condition showing stress concentration along the sharp edges**. Stresses at the contact surfaces are from 0.3 MPa to 15 MPa. Most of the green contour stress levels are 2.5 MPa.

Figure 9 shows the bone interface stress distribution for the second load case for the custom implant. The stress was evenly distributed across the two condyles, with minor stress concentrations along the medial edge of the medial condyle. Figure 10 shows the bone interface for the conventional implant under the third load condition. The stresses were concentrated along the sharp edges produced by the cutting operation.

The remaining FEA studies showed results that were consistent with the above cases.

## Discussion

The finite element analyses presented here were based on a perfect fit between the implant and the distal femur because Boolean operations were used to simulate the cuts. However, a perfect match of the contact surfaces is not very common when preparing them using hand tools and cutting guides. As reported earlier, the average contact surface is only about 50%, meaning that the actual loading of the joint was probably worse than what is reported here. It is anticipated that the true surface pressures on the standard implants would be higher. From the results above, it can be seen that a custom implant with a freeform bone interface would provide a more even stress distribution on the bone interface than the conventional femoral components. The FEA results for the conventional femoral components were consistent with earlier FEA studies and also with what has been seen in clinical cases. The stress concentrations along the sharp edges causes bone remodeling and increased bone density, while the areas between the sharp edges experience stress shielding that leads to bone resorption. This uneven bone remodeling is thought to lead to premature aseptic loosening, but it is also increases the complexity of a revision surgery due to missing bone.

The downside of using custom-designed implant components is the time and cost associated with the design, as well as the need for a surgical robot to perform the bone resection. However, with time and experience, the design of custom femoral components based on a CT scan could become highly streamlined, and the total time and cost could be reduced to a minimum. To fabricate custom-designed implant components at a reasonable cost has always been a problem and has discouraged this approach in the past. Recent developments in direct metal fabrication using Rapid Prototyping technologies can radically change the situation.

An Electron Beam Melting (EBM) machine is capable of fabricating 10–15 custom implant components in less than 15 hours in either titanium or cobalt-chromium at a reasonable cost [19]. The finishing operation is very similar to conventional implant fabrication and would not add significantly to the total cost. One advantage of fabricating a femoral implant component using the EBM technology is the ability to produce the porous bone ingrowth surface simultaneously. This will save time and cost otherwise associated with the sintering operation of titanium or cobalt-chromium beads, which is normally done in multiple steps and requires manual labor.

The need for an orthopedic robot to perform the cutting operation might be more difficult in the United States since such robots are still not FDA approved for orthopedic surgery. However, robotic systems are being used in European countries to assist with bone resection during implant surgeries for both hip and knee implantations. Soon such robotic systems might become FDA approved in the United States and readily available to orthopedic surgeons. These robots can easily produce the freeform bone-implant interface using a rotating mill cutter that would produce an almost perfect fit between the bone and the implant.

We recognize that the proposed custom implant system is not for every patient but can be applied to younger patients and those who have a more active lifestyle and will therefore depend on the implant for a long time. It is anticipated that custom-designed implants will increase the longevity and that the added cost can be justified for these younger, more active patients. With the recent improvements of direct metal fabrication systems, it is possible that the fabrication costs of custom implant components can be reduced to that of conventional implants, which would make custom-designed implants attractive even for older patients. If only custom implant components were used in combination with orthopedic robots, the current inventory of implant components held by hospitals could be eliminated. Further, the costs associated with purchasing, maintaining, and storing implant surgical instruments and trial components could be eliminated as well.

## Conclusion

The proposed custom femoral component design has the following advantages compared with a conventional femoral component. (i) Since the articulating surface closely mimics the shape of the distal femur, there is no need for resurfacing of the patella or gait change. (ii) Owing to the resulting stress distribution, bone remodeling is even and the risk of premature loosening might be reduced. (iii) Because the bone-implant interface can accommodate anatomical abnormalities at the distal femur, the need for surgical interventions and fitting of filler components is reduced. (iv) Given that the bone-implant interface is customized, about 40% less bone must be removed. The primary disadvantages are the time and cost required for the design and the possible need for a surgical robot to perform the bone resection. These disadvantages may be eliminated by the use of rapid prototyping technologies, especially the use of Electron Beam Melting technology for quick and economical fabrication of custom implant components.

## Competing interests

The author(s) declare that they have no competing interests.

## Authors' contributions

OLAH designed the study, carried out the analysis, and drafted the manuscript. YAH and JFN supervised the background work leading to the study. All authors read and approved the final manuscript.

## Pre-publication history

The pre-publication history for this paper can be accessed here:


